# Supplementary material for: Role and mechanism of NCAPD3 in promoting malignant behaviors in gastric cancer
Source: Front Pharmacol. 2024 Apr 22;15:1341039. doi: 10.3389/fphar.2024.1341039 (PMC11070777; doi:10.3389/fphar.2024.1341039)
Supplement: Supplementary file 11 [file DataSheet2.ZIP › GSEA/GO_CC/my_analysis.Gsea.1599461408869/GO_SUPRAMOLECULAR_POLYMER.html]

Details for gene set GO\_SUPRAMOLECULAR\_POLYMER[GSEA]

|  || Dataset | filtered\_dataset.sample\_info.cls#WT\_versus\_NCAPD3\_MUT |
| Phenotype | sample\_info.cls#WT\_versus\_NCAPD3\_MUT |
| Upregulated in class | NCAPD3\_MUT |
| GeneSet | GO\_SUPRAMOLECULAR\_POLYMER |
| Enrichment Score (ES) | -0.28245828 |
| Normalized Enrichment Score (NES) | -1.9703151 |
| Nominal p-value | 0.0 |
| FDR q-value | 0.03878629 |
| FWER p-Value | 0.131 |
Table: GSEA Results Summary

  

Fig 1: Enrichment plot: GO\_SUPRAMOLECULAR\_POLYMER      
 Profile of the Running ES Score & Positions of GeneSet Members on the Rank Ordered List

  

| SYMBOL | TITLE | RANK IN GENE LIST | RANK METRIC SCORE | RUNNING ES | CORE ENRICHMENT || 1 | 342184 | FMN1 | 9 | 1.110 | 0.0192 | No |
| 2 | 7170 | TPM3 | 24 | 1.018 | 0.0325 | No |
| 3 | 10513 | APPBP2 | 28 | 1.000 | 0.0537 | No |
| 4 | 9759 | HDAC4 | 46 | 0.925 | 0.0626 | No |
| 5 | 23322 | RPGRIP1L | 60 | 0.871 | 0.0732 | No |
| 6 | 51512 | GTSE1 | 127 | 0.774 | 0.0420 | No |
| 7 | 51174 | TUBD1 | 153 | 0.730 | 0.0404 | No |
| 8 | 7248 | TSC1 | 158 | 0.718 | 0.0542 | No |
| 9 | 9456 | HOMER1 | 198 | 0.680 | 0.0409 | No |
| 10 | 22948 | CCT5 | 215 | 0.663 | 0.0445 | No |
| 11 | 54801 | HAUS6 | 219 | 0.660 | 0.0577 | No |
| 12 | 324 | APC | 281 | 0.607 | 0.0263 | No |
| 13 | 4281 | MID1 | 294 | 0.595 | 0.0312 | No |
| 14 | 10426 | TUBGCP3 | 402 | 0.535 | -0.0362 | No |
| 15 | 259266 | ASPM | 411 | 0.531 | -0.0298 | No |
| 16 | 805 | CALM2 | 423 | 0.518 | -0.0259 | No |
| 17 | 801 | CALM1 | 424 | 0.518 | -0.0138 | No |
| 18 | 23242 | COBL | 426 | 0.516 | -0.0025 | No |
| 19 | 23116 | FAM179B | 482 | 0.486 | -0.0322 | No |
| 20 | 27229 | TUBGCP4 | 556 | 0.442 | -0.0764 | No |
| 21 | 84948 | TIGD5 | 619 | 0.413 | -0.1130 | No |
| 22 | 6905 | TBCE | 652 | 0.399 | -0.1276 | No |
| 23 | 23093 | TTLL5 | 684 | 0.386 | -0.1417 | No |
| 24 | 5311 | PKD2 | 708 | 0.372 | -0.1502 | No |
| 25 | 203068 | TUBB | 736 | 0.358 | -0.1620 | No |
| 26 | 10726 | NUDC | 754 | 0.346 | -0.1666 | No |
| 27 | 57619 | SHROOM3 | 783 | 0.331 | -0.1798 | No |
| 28 | 1605 | DAG1 | 823 | 0.304 | -0.2018 | No |
| 29 | 84823 | LMNB2 | 833 | 0.293 | -0.2017 | No |
| 30 | 6385 | SDC4 | 871 | -0.282 | -0.2227 | No |
| 31 | 667 | DST | 886 | -0.303 | -0.2261 | No |
| 32 | 27230 | SERP1 | 892 | -0.306 | -0.2227 | No |
| 33 | 9181 | ARHGEF2 | 902 | -0.326 | -0.2218 | No |
| 34 | 23710 | GABARAPL1 | 913 | -0.341 | -0.2213 | No |
| 35 | 301 | ANXA1 | 914 | -0.342 | -0.2133 | No |
| 36 | 84056 | KATNAL1 | 917 | -0.346 | -0.2067 | No |
| 37 | 4052 | LTBP1 | 928 | -0.353 | -0.2060 | No |
| 38 | 2317 | FLNB | 936 | -0.360 | -0.2028 | No |
| 39 | 8881 | CDC16 | 963 | -0.383 | -0.2133 | No |
| 40 | 23499 | MACF1 | 989 | -0.398 | -0.2226 | No |
| 41 | 7277 | TUBA4A | 997 | -0.404 | -0.2184 | No |
| 42 | 83896 | KRTAP3-1 | 1006 | -0.408 | -0.2149 | No |
| 43 | 23046 | KIF21B | 1034 | -0.433 | -0.2249 | No |
| 44 | 81631 | MAP1LC3B | 1060 | -0.452 | -0.2331 | No |
| 45 | 7168 | TPM1 | 1086 | -0.465 | -0.2409 | No |
| 46 | 6795 | AURKC | 1100 | -0.477 | -0.2394 | No |
| 47 | 7074 | TIAM1 | 1143 | -0.508 | -0.2589 | No |
| 48 | 1540 | CYLD | 1162 | -0.530 | -0.2600 | No |
| 49 | 1282 | COL4A1 | 1165 | -0.534 | -0.2490 | No |
| 50 | 3887 | KRT81 | 1168 | -0.536 | -0.2380 | No |
| 51 | 3866 | KRT15 | 1214 | -0.589 | -0.2578 | No |
| 52 | 114907 | FBXO32 | 1248 | -0.625 | -0.2679 | Yes |
| 53 | 63971 | KIF13A | 1256 | -0.645 | -0.2580 | Yes |
| 54 | 55638 | SYBU | 1275 | -0.663 | -0.2560 | Yes |
| 55 | 286 | ANK1 | 1284 | -0.672 | -0.2463 | Yes |
| 56 | 51175 | TUBE1 | 1286 | -0.677 | -0.2312 | Yes |
| 57 | 100820829 | MYZAP | 1291 | -0.682 | -0.2182 | Yes |
| 58 | 7414 | VCL | 1308 | -0.712 | -0.2136 | Yes |
| 59 | 3892 | KRT86 | 1313 | -0.724 | -0.1996 | Yes |
| 60 | 57553 | MICAL3 | 1318 | -0.732 | -0.1855 | Yes |
| 61 | 6262 | RYR2 | 1323 | -0.741 | -0.1712 | Yes |
| 62 | 6509 | SLC1A4 | 1330 | -0.752 | -0.1581 | Yes |
| 63 | 10611 | PDLIM5 | 1335 | -0.758 | -0.1434 | Yes |
| 64 | 80709 | AKNA | 1351 | -0.795 | -0.1360 | Yes |
| 65 | 4622 | MYH4 | 1355 | -0.811 | -0.1193 | Yes |
| 66 | 3860 | KRT13 | 1367 | -0.847 | -0.1078 | Yes |
| 67 | 5630 | PRPH | 1371 | -0.885 | -0.0893 | Yes |
| 68 | 6840 | SVIL | 1377 | -0.916 | -0.0717 | Yes |
| 69 | 10397 | NDRG1 | 1380 | -0.925 | -0.0516 | Yes |
| 70 | 6711 | SPTBN1 | 1381 | -0.925 | -0.0299 | Yes |
| 71 | 8867 | SYNJ1 | 1387 | -0.979 | -0.0108 | Yes |
| 72 | 493 | ATP2B4 | 1401 | -1.166 | 0.0067 | Yes |
Table: GSEA details [plain text format]

  

Fig 2: GO\_SUPRAMOLECULAR\_POLYMER      
 Blue-Pink O' Gram in the Space of the Analyzed GeneSet

  

Fig 3: GO\_SUPRAMOLECULAR\_POLYMER: Random ES distribution      
 Gene set null distribution of ES for **GO\_SUPRAMOLECULAR\_POLYMER**
